# Short-Time Infrequent Metadynamics for Improved Kinetics Inference

**DOI:** 10.1021/acs.jctc.4c00170

**Published:** 2024-04-26

**Authors:** Ofir Blumer, Shlomi Reuveni, Barak Hirshberg

**Affiliations:** † School of Chemistry, Tel Aviv University, Tel Aviv 6997801, Israel; ‡ The Center for Computational Molecular and Materials Science, Tel Aviv University, Tel Aviv 6997801, Israel; § The Center for Physics and Chemistry of Living Systems, Tel Aviv University, Tel Aviv 6997801, Israel

## Abstract

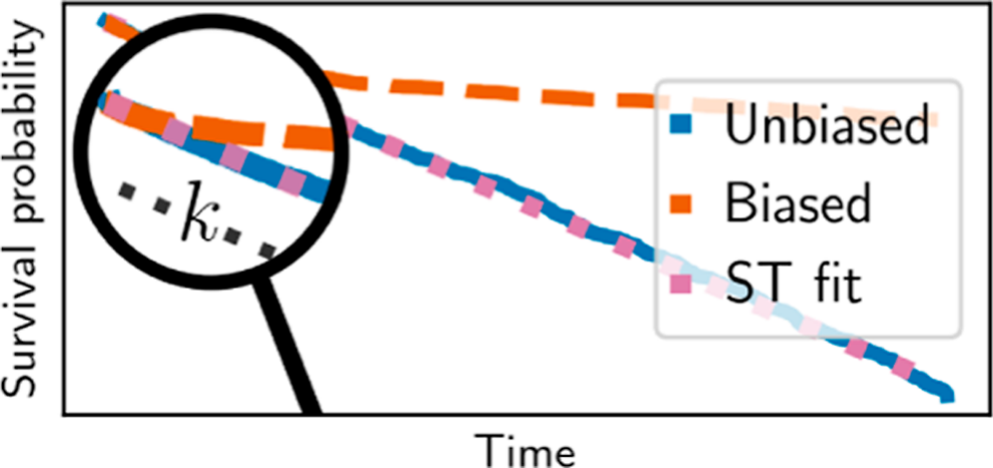

Infrequent Metadynamics is a popular method to obtain the rates of long time-scale processes from accelerated simulations. The inference procedure is based on rescaling the first-passage times of the Metadynamics trajectories using a bias-dependent acceleration factor. While useful in many cases, it is limited to Poisson kinetics, and a reliable estimation of the unbiased rate requires slow bias deposition and prior knowledge of efficient collective variables. Here, we propose an improved inference scheme, which is based on two key observations: (1) the time-independent rate of Poisson processes can be estimated using short trajectories only. (2) Short trajectories experience minimal bias, and their rescaled first-passage times follow the unbiased distribution even for relatively high deposition rates and suboptimal collective variables. Therefore, by basing the inference procedure on short time scales, we obtain an improved trade-off between speedup and accuracy at no additional computational cost, especially when employing suboptimal collective variables. We demonstrate the improved inference scheme for a model system and two molecular systems.

## Introduction

Molecular dynamics (MD) simulations are widely used to study complex systems at the microscopic level. Their atomic resolution allows evaluating thermodynamic and kinetic properties, but it also limits the accessible time scales.^1−4^ Therefore, long time-scale processes, such as protein folding or crystal nucleation, are almost never studied using brute-force, long simulations.^5^ Instead, enhanced sampling methods are usually employed.

Various methods were developed to study long time-scale processes through MD simulations. Some use a series of short simulations to sample the unbiased kinetics, such as milestoning,^6,7^ Markov state models,^8,9^ stochastic resetting (SR),^10,11^ and many others. Another approach is to introduce an external bias potential, enhancing the sampling along a low-dimensional collective variable (CV) space. The chosen CVs are usually slow modes that can distinguish between metastable states.^1−5^ Methods following this approach include umbrella sampling,^12,13^ conformational flooding,^14^ adiabatic free-energy dynamics,^15−17^ on-the-fly probability enhanced sampling (OPES),^18,19^ Metadynamics (MetaD),^3,20,21^ and Hyperdynamics.^122^

Here, we focus on infrequent MetaD (iMetaD), a method to extract unbiased kinetics from accelerated MetaD simulations. In iMetaD, several biased trajectories are initiated and stopped after a first-passage criterion is fulfilled. The first-passage time (FPT) of each trajectory is then rescaled by an acceleration factor that depends on the external bias deposited along the trajectory.^1,3^ The method assumes that the underlying process obeys Poisson kinetics, and the unbiased rate is estimated by fitting the rescaled FPTs to an exponential distribution.^2^

The key assumption of iMetaD is that no bias is deposited near the transition state.^1,5^ This assumption fails for high bias deposition rates or suboptimal CVs that lead to hysteresis and bias overdeposition.^2,5,22^ Unfortunately, finding good CVs in complex systems remains a great challenge,^23,24^ despite recent developments.^25−132^ Thus, to improve the inference, one is usually forced to limit the bias deposition rate, but this also reduces the acceleration, resulting in a trade-off between speedup and accuracy.^4,11,22^

The reliability of iMetaD is usually assessed through a procedure suggested by Salvalaglio et al.^2,5^ A Kolmogorov–Smirnov (KS) test^32^ is performed to accept or reject the hypothesis that the rescaled FPTs are taken from an exponential distribution. The results are considered reliable for a *p*-value greater than 0.05 (though there are examples of erroneous results that pass the test^135,136^) and the unbiased mean FPT (MFPT) is then estimated as the mean of the exponential fit to the rescaled FPT distribution. Trajectories with hysteresis or overdeposition contribute unrealistically long rescaled FPTs, leading to distributions that are broader than exponential and failure of the KS test. In this paper, we propose an improved inference scheme which deals with this prevalent problem, extending the range of applicability of iMetaD. We also compare our method with the Kramers time-dependent rate (KTR) method that was recently introduced with a similar goal in mind.^4,5^

Our scheme relies on two key observations: (1) Since exponential distributions are characterized by a single parameter (their time-independent kinetic rate), short simulations, showing a single transition each, are sufficient to estimate the full distribution reliably. (2) The rescaling procedure of iMetaD is more reliable for short trajectories, experiencing minimal bias. This can be seen from the rescaled FPT distribution, which often follows the unbiased distribution at short times, even when using high bias deposition rates or suboptimal CVs. The improved scheme is inspired by our previous work, combining MetaD with SR.^11^ Previously, we showed that SR provides enriched sampling of short time scales, leading to an improved trade-off between speedup and accuracy. Interestingly, these observations are not limited to simulations with SR.

We next show how to exploit our observations to build an improved kinetic inference scheme for iMetaD simulations. We refer to this scheme as short-time iMetaD (ST-iMetaD). ST-iMetaD extends the applicability of iMetaD to higher bias deposition rates and suboptimal CVs, reducing the prediction errors by orders of magnitude in comparison to the standard procedure. We demonstrate its advantages in three systems of increasing complexity: the two-dimensional Wolfe–Quapp potential, alanine dipeptide in vacuum, and the unfolding of the chignolin miniprotein in water.

## ST-iMetaD Scheme

We present ST-iMetaD through the example of the Wolfe–Quapp potential. It is a two-state model previously used to study the performance of suboptimal CVs.^22,23^ Its exact form is given in the Methods section, as are all simulation details. We first performed 1000 brute-force, standard MD simulations to obtain the unbiased FPT distribution and found the MFPT to be ∼110 ns. A KS test confirmed that the unbiased distribution is exponential (*p*-value of 0.81).

**Figure 1:**
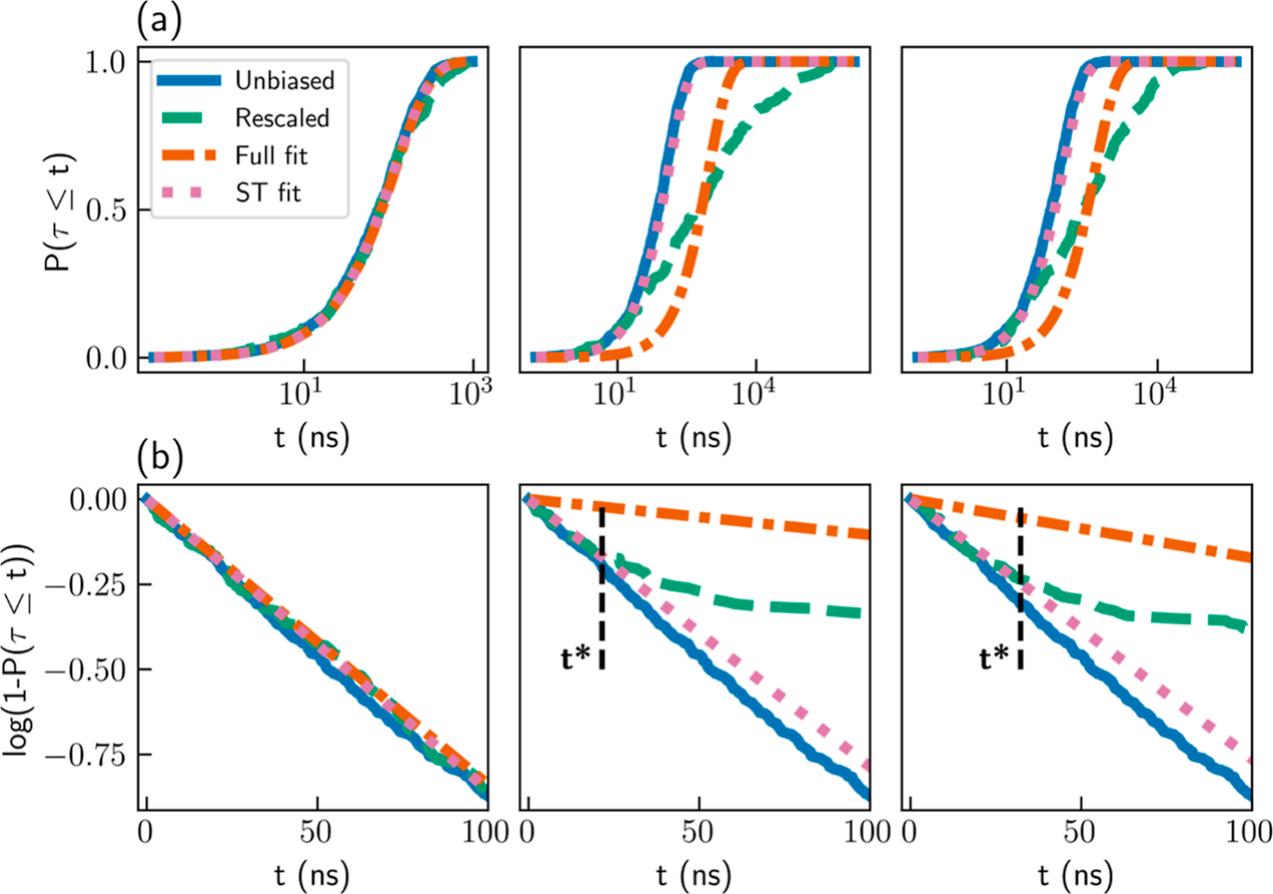
(a) CDF profiles and (b) survival functions for simulations of the Wolfe–Quapp potential. Results for unbiased FPTs (blue solid lines), rescaled FPTs (dashed green lines), exponential fits to the rescaled CDF in the entire range (orange dashed–dotted lines), and linear fits to the survival functions at *t* ≤ t* (pink dotted lines). Results are shown for iMetaD simulations using a good CV and a bias deposition rate of 10 ns^–1^ (left), a good CV and a bias deposition rate of 1000 ns^–1^ (middle), and a suboptimal CV and a bias deposition rate of 200 ns^–1^ (right). The black dashed lines mark *t* = t*.

Next, we performed 200 iMetaD simulations with a good CV and a slow bias deposition rate of 10 ns^–1^, updating the bias every 10^5^ timesteps. The quality of the CV was proved using a committor analysis^33,34^ (see the Supporting Information for details). With this choice of parameters, we expect the underlying assumptions of iMetaD to be valid and the original inference scheme to be accurate. Indeed, the MFPT estimated through the standard inference procedure is 119 ns, in good agreement with the true value. A *p*-value of 0.25 confirms the reliability of the results. The left panel of Figure 1a shows the cumulative distribution function (CDF) *P*(τ ≤ *t*) for both the unbiased FPTs (blue solid line) and the rescaled FPTs (green dashed line). An exponential fit to the CDF of the rescaled FPTs is given in an orange dashed–dotted line. We find a good agreement between all three curves, showing that the standard inference procedure is adequate in this case.

We then performed iMetaD simulations using the same CV but with a higher bias deposition rate of 1000 ns^–1^ (every 1000 timesteps), which is expected to give poor inference due to hysteresis. The obtained MFPT, 953 ns, overestimates the true value by an order of magnitude, and the *p*-value of the KS test drops to 2 × 10^–11^, indicating that the results are unreliable. The middle panel of Figure 1a again shows the CDF for the unbiased and rescaled FPTs, as well as the exponential fit to the rescaled CDF. In this case, we find that the rescaled CDF is not exponential and clearly deviates from the unbiased CDF. Consequently, the exponential fit results in the wrong estimate of the rate and MFPT. Nevertheless, we find that the unbiased and rescaled CDFs are in very close agreement at short times. This is the first key observation of this work: short trajectories experience minimal bias, and thus their rescaled CDF reflects the correct statistics for small FPTs even at high bias deposition rates.

A similar phenomenon is observed when suboptimal CVs are used. We select a moderate bias deposition rate of 200 ns^–1^ and intentionally reduce the quality of the CV by rotating it at an angle θ = 56° relative to the good CV. The right panel of Figure 1a presents the resulting CDF profiles. Once again, the rescaled CDF is far from exponential (*p*-value of 7 × 10^–9^), and the MFPT is overestimated (579 ns). However, even though the rescaled CDF deviates from the unbiased CDF at long times, they match closely at short times.

When employing iMetaD, it is common practice to present the rescaled CDF profile, which is used for the KS test.^2,4,5^ However, for the rest of this paper, it would be more convenient to examine the survival function, 1 – *P*(τ ≤ *t*), since its logarithm decays linearly for exponential distributions. Figure 1b gives log[1 – *P*(τ ≤ *t*)] at *t* ≤ 100 ns for the unbiased FPTs (blue solid lines) and the rescaled FPTs (green dashed lines) of the simulations presented in Figure 1a. The unbiased survival function decays linearly, as expected. When the assumptions of iMetaD hold (left panel), the rescaled survival function closely follows the unbiased one. When they break (middle and right panels), the rescaled survival function matches the unbiased one up to some finite time, *t* = t*, and decays slower at *t* > t*. As a result, the exponential fits to the rescaled data (orange dashed–dotted lines) decay much slower than the unbiased curves. This explains the overestimated MFPT values. Note that we fit the rescaled survival function for all *t* but only display *t* ≤ 100 ns.

We improve the inference by fitting a linear function *S*(*t*) = −*kt* to the rescaled survival function only at *t* ≤ t* (dotted pink lines in Figure 1). We then estimate the MFPT as *k*^–1^. Notice that we only fit a single parameter *k* as the survival function must fulfill log[1 – *P*(τ ≤ *t* = 0)] = 0 due to normalization. In all three cases, we find that these short-time fits are closer to the unbiased survival function and therefore lead to an improved estimate of the MFPT, as we show below. First, we explain how to choose an adequate value of t*.

We use the Pearson correlation coefficient *R*, which quantifies the quality of the linear fit to the survival function. Practically, we perform multiple fittings, with different choices of t*, and select the fit resulting in the highest value of *R*^2^. Our results show that this approach correctly identifies reasonable values of t*, such that the rescaled survival functions match the unbiased ones at *t* ≤ t*. Specifically, for a good CV and a low bias deposition rate, where the results are reliable, we find t* = 148 ns. For the same CV and a high bias deposition rate and for a poor CV and a moderate rate, we obtain lower values, t* = 21 ns and t* = 32 ns, respectively (black dashed lines in Figure 1b).

To summarize our method, the main modification to the original inference scheme is that instead of fitting an exponential distribution to all of the data, as is customary, we limit the analysis to short time scales. We perform a series of linear fits to the logarithm of the survival function at times *t* ≤ t*, with different choices of t*. The parameter *k* of the best fit is taken as the kinetic rate, and the MFPT is estimated as *k*^–1^. This enables accurate estimations of the MFPT, even with frequent bias deposition or a suboptimal CV.

## Results and Discussion

### Wolfe–Quapp Potential

**Figure 2:**
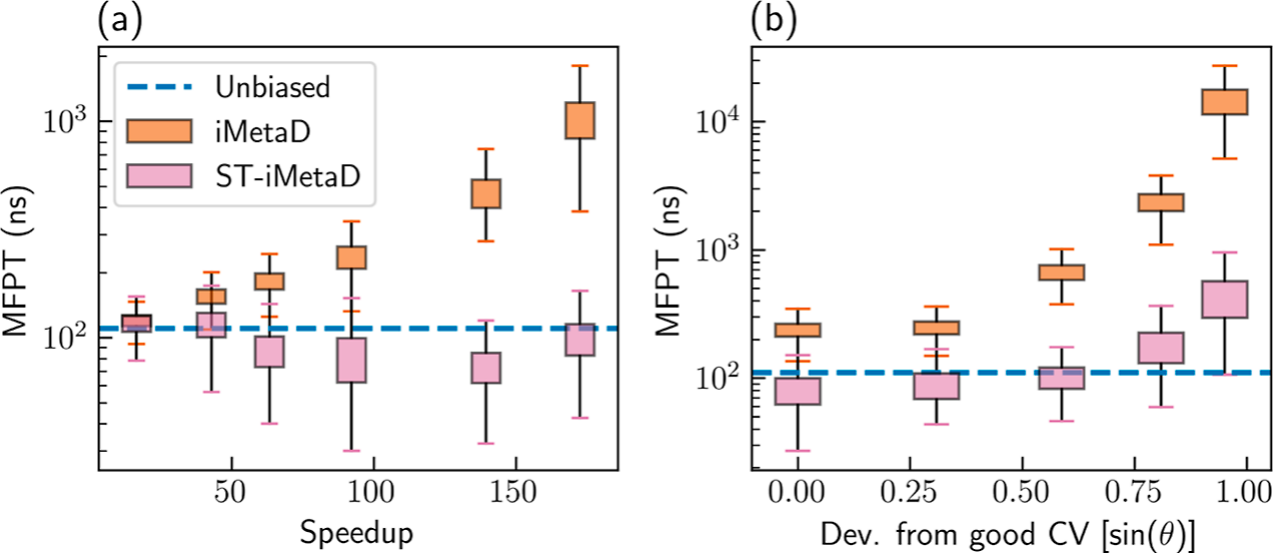
(a) Estimated MFPTs as a function of the speedup for the Wolfe–Quapp potential using a good CV and different bias deposition rates from 10 to 1000 ns^–1^. (b) Estimated MFPTs for a bias deposition rate of 200 ns^–1^ and different choices of CV. The blue lines mark the unbiased MFPT value. We employed either standard iMetaD (orange) or ST-iMetaD (pink). The boxes show the range between the first and third quartiles and the whiskers show extreme values within 1.5 IQR below and above these quartiles.

We first demonstrate the advantages of ST-iMetaD using the Wolfe–Quapp potential, showing that we can use higher bias deposition rates, providing higher speedups with minimal penalty to the inference accuracy. We define the speedup as the ratio between the unbiased MFPT and the MFPT from the biased simulations without rescaling. We ran a total of 1000 trajectories and performed a bootstrapping analysis on 1000 randomly sampled sets, each containing 200 samples. Figure 2a shows the estimated MFPT as a function of the speedup using a good CV and different bias deposition rates in the range of 10–1000 ns^–1^. The boxes show the range between the first and third quartiles (interquartile range, IQR), and the whiskers show extreme values within 1.5 IQR below and above these quartiles. When employing standard iMetaD (orange), the estimated MFPT increases with speedup, reaching values about an order of magnitude larger than the true value at high speedups. On the other hand, when employing ST-iMetaD (pink), the estimations remain close to the true value for all speedups.

Our scheme also improves the inference from simulations performed with suboptimal CVs. For a fixed bias deposition rate of 200 ns^–1^, we gradually reduce the quality of the CV by rotating it with respect to a good CV. Figure 2b shows that the estimated MFPT increases as the quality of the CV decreases for both inference schemes. However, the deviation from the true value is much smaller for ST-iMetaD. The errors remain within an order of magnitude of the true value, in comparison to more than 2 orders of magnitude for standard iMetaD, even for very poor CVs. In the Supporting Information, we also provide a detailed comparison with the KTR method.^4^

**Figure 3:**
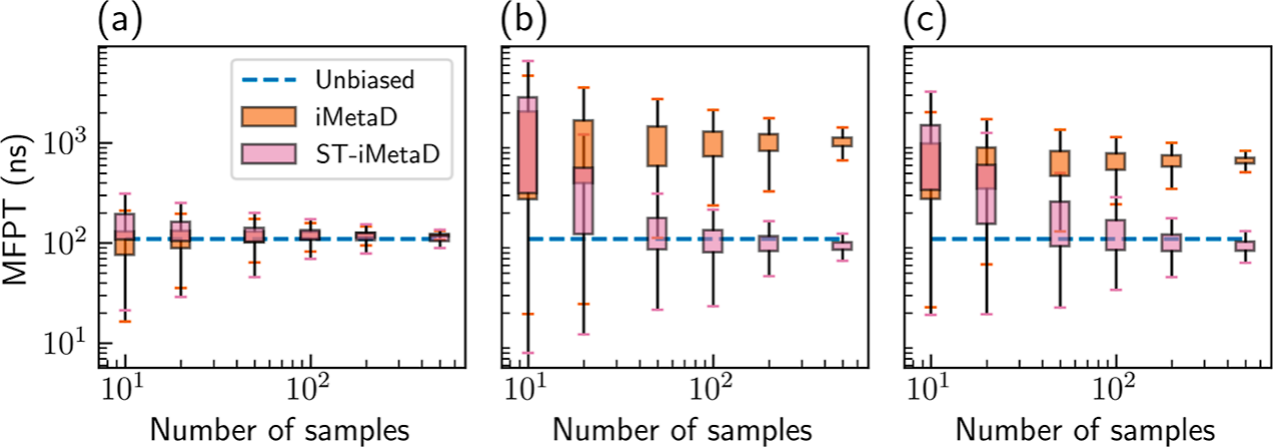
Estimated MFPT as a function of the number of sampled trajectories in each bootstrapping batch using iMetaD (orange) or ST-iMetaD (pink) for simulations of the Wolfe–Quapp potential using a (a) good CV and a bias deposition rate of 10 ns^–1^, a (b) good CV and a bias deposition rate of 1000 ns^–1^, and a (c) suboptimal CV and a bias deposition rate of 200 ns^–1^. The unbiased MFPT is given in blue dashed lines. The boxes show the range between the first and third quartiles and the whiskers show extreme values within 1.5 IQR below and above these quartiles.

We tested the sensitivity of ST-iMetaD to the number of sampled trajectories. For each column in Figure 1, we ran a total of 1000 trajectories and performed a bootstrapping analysis on 1000 randomly sampled sets, each containing 10, 20, 50, 100, 200, and 500 samples. Figure 3 shows the estimated MFPT using either iMetaD (orange) or ST-iMetaD (pink) as a function of the number of samples. In Supporting Information Figure S2, we also plot the dependence of t* on the batch size. We find that iMetaD has a systematic error that is almost independent of the number of samples, while both the systematic and statistical errors of ST-iMetaD diminish with additional data. With limited data of 10 or 20 samples, ST-iMetaD gives results comparable to those of iMetaD, but 50 samples are already sufficient for a major improvement. For the remainder of the paper, we report results obtained with bootstrapping sets of 200 random samples. Equivalent figures with smaller sample sizes are provided in the Supporting Information.

### Alanine Dipeptide

We next apply ST-iMetaD in two molecular systems, starting with the well-studied example of alanine dipeptide in vacuum. Alanine dipeptide has two stable conformers, *C*_7eq_ and *C*_7ax_, and is usually described by two dihedral angles, ϕ and ψ, with ϕ serving as a good CV and ψ as a suboptimal one^1,2,18,22^ (see ref (1) for definitions of conformers and angles). Transitions from the *C*_7eq_ conformer to the *C*_7ax_ conformer have an estimated MFPT of ∼3.5 μs (see Supporting Information for more details). We performed MetaD simulations with bias deposition rates in the range of 20 to 1000 ns^–1^ and either the ϕ or ψ angle as a CV. Full simulation details are given in the Methods section.

**Figure 4:**
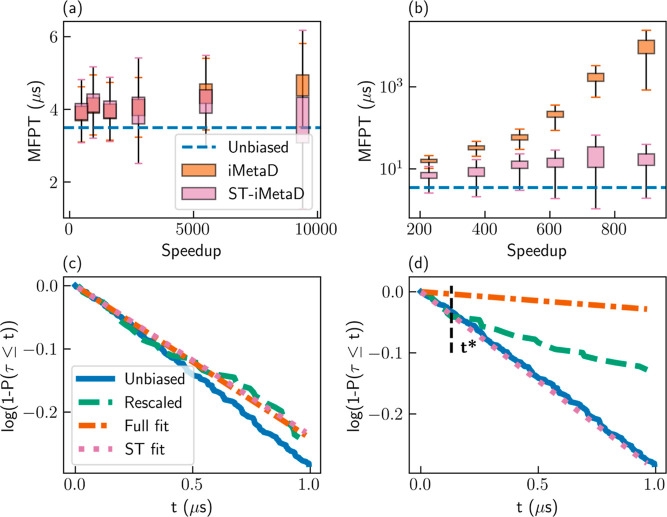
Upper row: estimation of the MFPT as a function of speedup for the *C*_7eq_–*C*_7ax_ conformer transition of alanine dipeptide in vacuum. Simulations using either the (a) ϕ angle or (b) ψ angle as a CV, with iMetaD (orange) or ST-iMetaD (pink). The boxes show the range between the first and third quartiles and the whiskers show extreme values within 1.5 IQR below and above these quartiles. The blue dashed lines show the unbiased MFPT. Lower row: survival functions log[1 – *P*(τ ≤ *t*)] at *t* ≤ 1 μs for unbiased simulations (blue solid lines) and rescaled iMetaD simulations (green dashed lines), biasing either the (c) ϕ angle or (d) ψ angle, with a bias deposition rate of 50 ns^–1^. Additional lines show exponential fits to the rescaled CDF in the entire range (orange dashed–dotted lines) and linear fits to the survival functions at *t* ≤ t* (pink dotted lines). The black dashed line marks *t* = t*.

Figure 4a shows the estimated MFPT as a function of the speedup for simulations biasing the ϕ angle through the original iMetaD scheme (orange) and ST-iMetaD (pink). The unbiased MFPT is given in a dashed blue line. The two schemes provide similar, very accurate estimations, even with frequent bias deposition. This confirms that ST-iMetaD is consistent with standard iMetaD, when the latter is reliable.

On the other hand, when ψ, a suboptimal CV, is employed, we find a major difference between the schemes, as demonstrated in Figure 4b. With standard iMetaD, the estimated MFPT rapidly increases with speedup (notice the logarithmic scale), reaching errors of more than 3 orders of magnitude. However, with ST-iMetaD, we obtain estimations within up to about an order of magnitude from the true value for all speedups.

We validate the underlying assumptions of ST-iMetaD by examining the survival functions. Panels (c,d) of Figure 4 show the survival functions for the unbiased FPT distribution (solid blue lines), the rescaled FPT distributions (dashed green lines), the fit of iMetaD (dashed–dotted orange lines), and the fit of ST-iMetaD (pink dotted lines). Results are shown for simulations with a moderate bias deposition rate of 50 ns^–1^, which is standard for iMetaD.^1,2^ Using the ϕ angle as a CV, the rescaled survival function decays at a rate similar to that of the unbiased one [panel (c)]. We determine t* = 12.6 μs using the procedure described above, and the two fits coincide. Using the ψ angle as a CV, the rescaled survival function quickly deviates from the unbiased one but is accurate at short times [panel (d)]. We correctly determine t* = 0.13 μs, and we obtain an MFPT estimation of 3.4 μs, improving by an order of magnitude over standard iMetaD.

### Chignolin Miniprotein

We close this paper with a more complex example: the unfolding of chignolin in explicit water (simulations of 5889 atoms). Chignolin is a miniprotein composed of 10 amino acids,^35^ previously used to benchmark enhanced sampling methods.^11,22,36−38^ A linear combination of six interatomic contacts, optimized via harmonic linear discriminant analysis (HLDA) by Mendels et al.,^25^ serves as a good CV. The radius of gyration (*R*_g_) and the C-alpha root-mean-square deviation from a folded configuration (RMSD) serve as examples of suboptimal CVs. All simulation details are provided in the Methods section.

**Figure 5:**
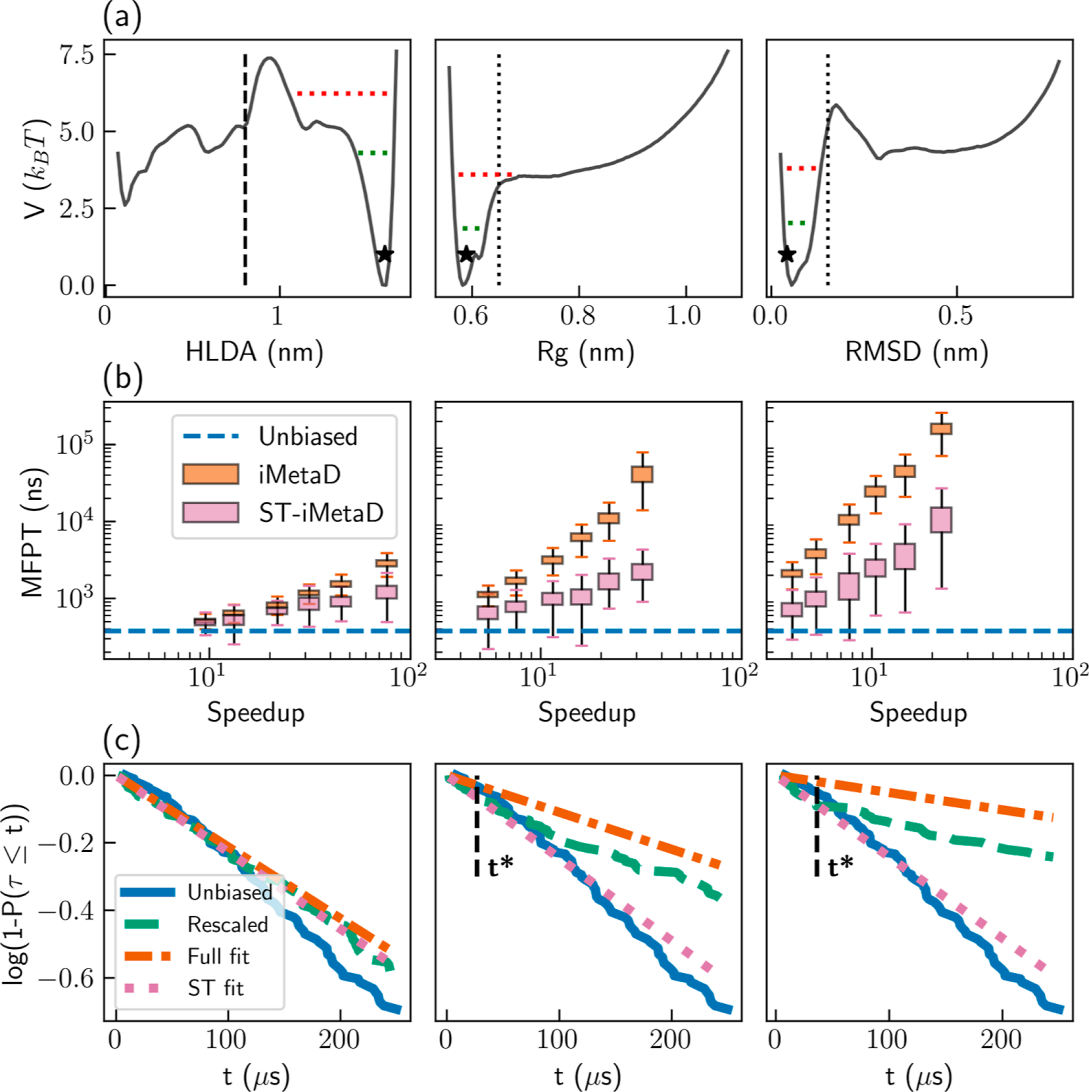
Results for the simulation of solvated chignolin using the HLDA-based CV (left), the radius of gyration (middle), and the C-alpha RMSD (right). (a) FESs. The vertical dashed line marks the first-passage criterion and the dotted vertical lines mark the average radius of gyration and C-alpha RMSD at first-passage events. The horizontal green and red lines highlight average maximal bias heights at t* and 10t*, respectively. The black stars mark the values of the CVs at the initial folded configuration. (b) Estimated MFPTs obtained with standard iMetaD (orange) and ST-iMetaD (pink). The boxes show the range between the first and third quartiles and the whiskers show extreme values within 1.5 IQR below and above these quartiles. The blue dashed lines show the unbiased MFPT. (c) Survival functions for unbiased FPTs (blue solid lines), rescaled FPTs of simulations with a bias deposition rate of 1 ns^–1^(dashed green lines), exponential fits to the rescaled CDF in the entire range (orange dashed–dotted lines), and linear fits to the survival function at *t* ≤ t* (pink dotted lines). The black dashed lines show the estimated t*.

Figure 5a gives the free-energy surfaces (FES) along all CVs, obtained from umbrella sampling simulations (see the Methods section for details). The values of the CVs at the initial folded configuration are marked with black stars. We define the first-passage criterion as reaching a value < 0.8 nm for the HLDA-based CV (dashed black line). This process has an estimated MFPT of ∼376 ns (see Supporting Information for details). We note that the dynamics for reaching a stable unfolded state (HLDA < 0.2 nm) leads to an MFPT that is longer by an order of magnitude.^22^ However, since the underlying assumptions of iMetaD are valid for escaping a single energy well, we limit our discussion to overcoming the first energy barrier along the HLDA-based CV. In addition, we note that the suboptimal CVs do not have a second minimum in the FES for the stable unfolded state. Moreover, they do not fully distinguish between the unfolded and folded states. Therefore, even when biasing the suboptimal CVs, we used the value of the HLDA-based CV to determine the FPTs. The dotted lines in the middle and right panels of Figure 5a show the average values of those CVs when the first-passage criterion is fulfilled.

Figure 5b shows the estimated MFPT as a function of speedup using the different CVs, with bias deposition rates in the range of 1–50 ns^–1^. We observe trends similar to those in the previous examples. In all cases, we find that ST-iMetaD leads to a better trade-off between speedup and accuracy. Most notably, it is able to predict the MFPT rather successfully, even for deposition rates where the standard approach leads to large errors.

As with the former examples, we verify our assumptions by plotting the survival functions for the slowest bias deposition rate, 1 ns^–1^ (Figure 5c). For all CVs, the rescaled and unbiased results match at short times. The suboptimal CVs are associated with short t*, while the value for the good CV is out of the scope of the plot (425 ns).

As a final test, we estimate the average maximum bias deposited up to t* and present it as a green horizontal dotted line in Figure 5a. We find that it is lower than the barrier for both suboptimal and good CVs. This analysis provides insight into the onset of bias overdeposition. This is seen by looking at the average bias deposited at 10t*, marked by horizontal dotted red lines in Figure 5a, which are much closer to the barrier. This confirms that our procedure identifies the right t* within an order of magnitude.

## Conclusions

To summarize, we present ST-iMetaD—an improved inference scheme for iMetaD simulations. We find that the rescaled FPT distribution provides the correct short-time statistics, even for high bias deposition rates and suboptimal CVs. By focusing on these time scales, the time-independent rate of Poisson processes can be estimated reliably, resulting in a better trade-off between speedup and accuracy in predicting the unbiased MFPTs.

The benefits of ST-iMetaD are demonstrated for the Wolfe–Quapp potential and two molecular systems: an isolated alanine dipeptide molecule and chignolin in explicit water. It reduces the prediction errors by orders of magnitude, especially for simulations with frequent bias deposition or suboptimal CVs. As a result, our method significantly extends the range of applicability of iMetaD, though it will eventually also break for unrealistically high deposition rates or exceedingly bad CVs. The ST-iMetaD scheme can be applied in postprocessing of existing iMetaD data, with no additional cost in comparison to the standard approach, leading to improved accuracy. Furthermore, the inference scheme is not limited to iMetaD and could be applied to any enhanced sampling approach based on the iMetaD rescaling scheme, such as OPES flooding^22^ or variational flooding.^39,40^

## Methods

### Wolfe–Quapp Potential

Simulations of the Wolfe–Quapp potential were performed in the large-scale atomic/molecular massively parallel simulator (LAMMPS).^41^ We followed the motion of a single particle with a mass *m* = 40 a.u. The simulations were carried out in the canonical (*NVT*) ensemble at a temperature of 300 K using a Langevin thermostat.^42^ The integration time step was 1 fs and the friction coefficient was 0.01 fs^–1^. MetaD was implemented using PLUMED 2.7.1.^43−45^ We used a bias height of 0.5 *k*_B_*T*, a bias factor of 5, a bias width of σ = 0.1 nm, and a grid spacing of 0.01 nm.

**Figure 6:**
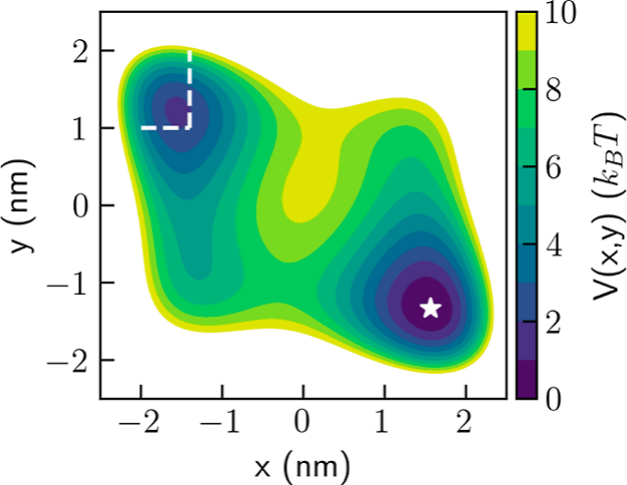
Wolfe–Quapp potential. The initial position is marked with a star and the target basin is marked with dashed lines.

The external potential was implemented in the LAMMPS input files. Its structure is as described in previous publications^22,23^ and is shown in Figure 6. The exact form used is given in eq 1, with the distance given in units of nm and the energy given in units of 1 *k*_B_*T*.
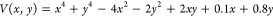
1

Simulations were initiated from the global minimum (*x* = 1.564, *y* = −1.334) nm, marked with a star in Figure 6, with velocities sampled from the Maxwell–Boltzmann distribution. All trajectories were stopped using the COMMITTOR command in PLUMED when reaching the second local minimum, defined as *x* < −1.4 nm ∧ *y* > 1.0 nm, denoted by dashed lines in Figure 6.

### Alanine Dipeptide

Simulations of alanine dipeptide in vacuum were performed in GROMACS 2019.6.^46^ We used input files by Bonomi and Bussi,^47^ implementing the AMBER99SB force field (FF). Simulations were performed in the *NVT* ensemble at a temperature of 300 K using a stochastic velocity rescaling thermostat.^48^ The integration time step was 2 fs. MetaD was implemented once again using PLUMED. We used a bias height of 0.5 *k*_B_*T*, a bias factor of 5, a bias width of σ = 0.25 rad, and a grid spacing of 0.01 rad. All trajectories were initiated in a fixed position at the *C*_7eq_ conformer and stopped using PLUMED when reaching 0.5 < ϕ < 1.5 rad. The stopping criterion was checked every 1 ps.

### Chignolin

Simulations of chignolin in water were performed using the same software as those for alanine dipeptide. We used input files by Ray et al.,^22^ available at PLUMED-NEST, the public repository of the PLUMED consortium,^45^ as plumID:22.031. We used the CHARMM22* FF^49^ for the protein and the CHARMM TIP3P FF^50^ for water. The thermodynamic ensemble, thermostat, and integration time step were the same as those employed for alanine dipeptide, but the temperature was higher, 340 K.

We used a bias height of 0.5 *k*_B_*T*, a bias factor of 5, and a grid spacing of 0.001 nm for all CVs. The bias width was 0.022, 0.005, and 0.006 nm for the HLDA, *R*_g_, and RMSD-based CVs, respectively. All trajectories were initiated from a fixed position and stopped using PLUMED when reaching *s* < 0.8 nm, with *s* being the HLDA-based CV. This stopping criterion was checked every 1 ps.

To construct the FES featured in Figure 4a, we performed 32, 100 ns long umbrella sampling simulations^12,13^ for each CV, with harmonic constraints centered at *s*_min_ + *i*Δ_s_, with *i* going from 0 to 31. We used *s*_min_ = 0.5, 6, and 0.25 Å and Δ_s_ = 0.5, 0.167, and 0.25 Å for the HLDA, *R*_g_, and RMSD-based CVs, respectively. The harmonic constant was *k* = 3 *k*_B_*T*Å^–2^ for all CVs. The value of the CV was saved every 1 ps, and the FES was constructed through the weighted histogram analysis method using the implementation of Grossfield.^51^

## Data Availability

Example input files, source data, and an example analysis script to perform ST-iMetaD are available in the GitHub repository: https://github.com/OfirBlumer/ST-iMetaD.

## Supporting Information Available

Details on the committor analysis and MFPT estimation, sensitivity of the results to the bootstrapping batch size, and comparison to the KTR method (PDF)
