# Supplementary material for: Short-Time Infrequent Metadynamics for Improved Kinetics Inference
Source: J Chem Theory Comput. 2024 Apr 26;20(9):3484–91. doi: 10.1021/acs.jctc.4c00170 (PMC11099961; doi:10.1021/acs.jctc.4c00170)
Supplement: Supplementary file 1 — ct4c00170_si_001.pdf [file ct4c00170_si_001.pdf]

Supporting Information: Short-Time Infrequent Metadynamics for Improved Kinetics Inference

Ofir Blumer,[†] Shlomi Reuveni,[†,‡,¶] and Barak Hirshberg[*,†,‡,¶]

†*School of Chemistry, Tel Aviv University, Tel Aviv 6997801, Israel.*

‡*The Center for Computational Molecular and Materials Science, Tel Aviv University, Tel Aviv 6997801, Israel.*

¶*The Center for Physics and Chemistry of Living Systems, Tel Aviv University, Tel Aviv 6997801, Israel.*

E-mail: hirshb@tauex.tau.ac.il

Committor analysis

Here we report the details of the committor analysis performed to estimate the quality of different collective variables (CVs) for the Wolfe-Quapp potential. Our procedure is based on that described by Peters.[S1] We defined two states, A at $x > 1.4\,nm$, $y < -1.0\,nm$ and B at $x < -1.4\,nm$, $y > 1.0\,nm$. For each examined CV $s(x, y)$, we first performed a $10\,ns$ long simulation initiated at $(x = 0, y = 0)\,nm$, restricted to $s \approx 0\,nm$ by a bias potential $V(s) = 80s^2$, with $V(s)$ in units of $1\,k_BT$ and s in nm. A thousand uncorrelated configurations were randomly sampled from the restricted trajectory, to serve as new initial configurations. Next, we sampled 100 trajectories for each initial configuration and obtained the fraction of trajectories that reached the target state B before state A, p_B.

Finally, we examined the histogram of p_B for all tested CVs. The CV showing the narrowest distribution around $p_B = 0.5$ best approximates the true committor and is regarded in the main text as the good CV. It is given by $s(x, y) = \cos\left(\frac{\pi}{9}\right) x - \sin\left(\frac{\pi}{9}\right) y$. Figure S1 shows the histograms of p_B for this CV and for $s(x, y) = x$, which is a poor CV, for comparison.

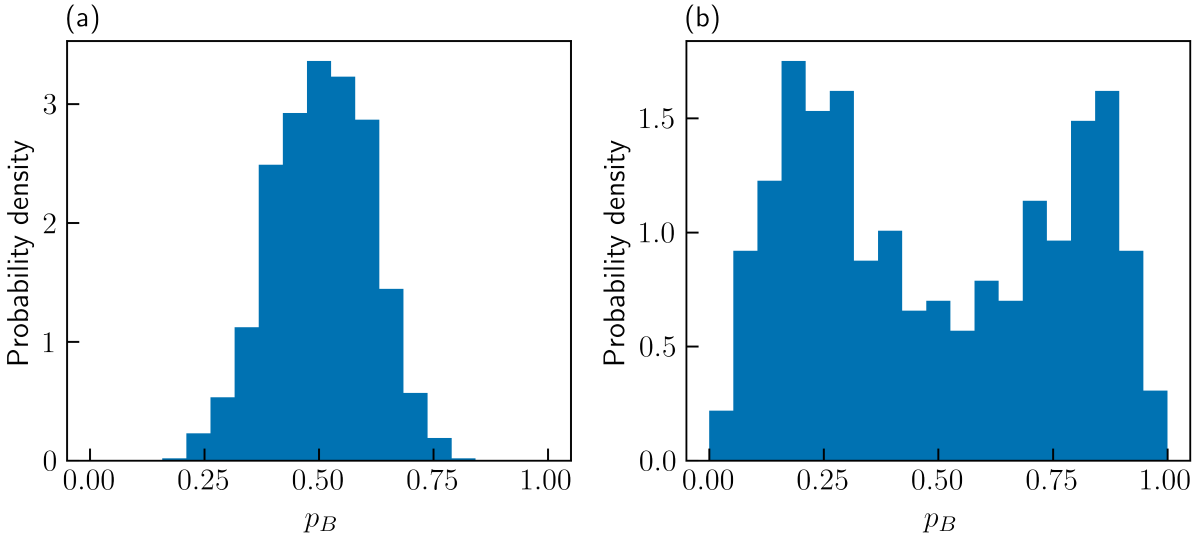

Figure S1: Histograms of p_B for (a) the good CV and (b) a poor CV.

Mean first-passage time estimation

Here we explain how the unbiased mean first-passage time (MFPT) values of the different processes were evaluated. For all systems, we first performed standard molecular dynamics simulations, that were stopped when the first-passage criterion was fulfilled. For the Wolfe-Quapp potential, all trajectories exhibited a first-passage. The MFPT was simply taken as the average first-passage time (FPT) of all trajectories. However, for both molecular systems, $\sim 7\%$ of the simulations did not show a first-passage event. Therefore, taking the MFPT of all trajectories would underestimate the true value.

Instead, we used the assumption that the FPT distribution is exponential. We made a linear fit to the logarithm of the survival function, and evaluated the true MFPT as $-k^{-1}$,

with k being the slope of the fit. For both systems, we obtained high coefficient of determination values, $R^2 > 0.995$, confirming that the underlying distributions are exponential.

Sensitivity of t^* to batch size

Here, we plot the values of t^* as a function of the bootstrapping batch size, for the systems shown in Figure 3 of the manuscript.

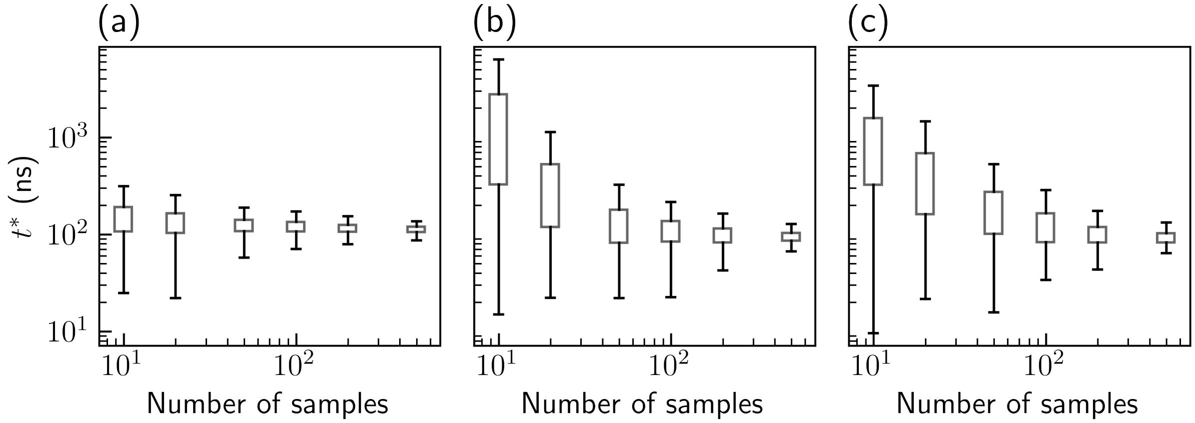

Figure S2: t^* as a function of the number of samples in each bootstrapping batch for simulations using (a) a good CV and bias deposition rate of $10\,ns^{-1}$, (b) a good CV and bias deposition rate of $1000\,ns^{-1}$, and (c) a suboptimal CV and bias deposition rate of $200\,ns^{-1}$. The boxes show the range between the first and third quartiles (interquartile range, IQR) and the whiskers show extreme values within 1.5 IQR below and above these quartiles.

Sensitivity of estimated MFPT to batch size

Here, we provide plots equivalent to Figure 2, Figure 4(a), Figure 4(b), and Figure 5(b) of the manuscript, with smaller bootstrapping batch sizes N.

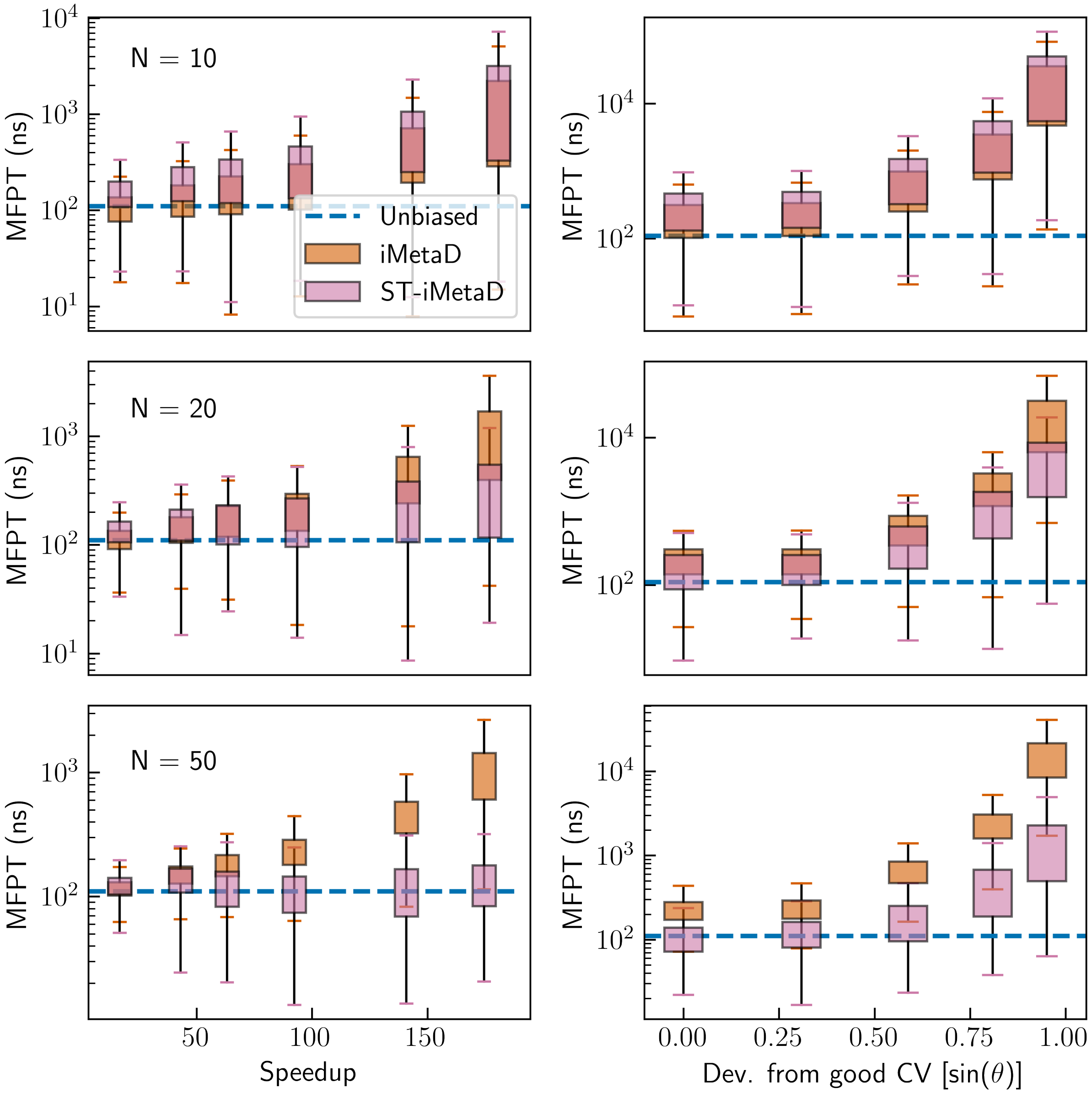

Figure S3: Results for the Wolfe-Quapp potential with sets of 10 (upper row), 20 (middle row), or 50 (bottom row) trajectories in each bootstrapping batch, using standard iMetaD (orange) or ST-iMetaD (pink). Left: Estimated MFPT as a function of speedup for simulations using a good CV and different bias deposition rates from 10 to 1000 ns^{-1}. Right: Estimated MFPTs for a bias deposition rate of 200 ns^{-1}, and different choices of CV. The boxes show the range between the first and third quartiles and the whiskers show extreme values within 1.5 IQR below and above these quartiles. The blue lines show the unbiased MFPT.

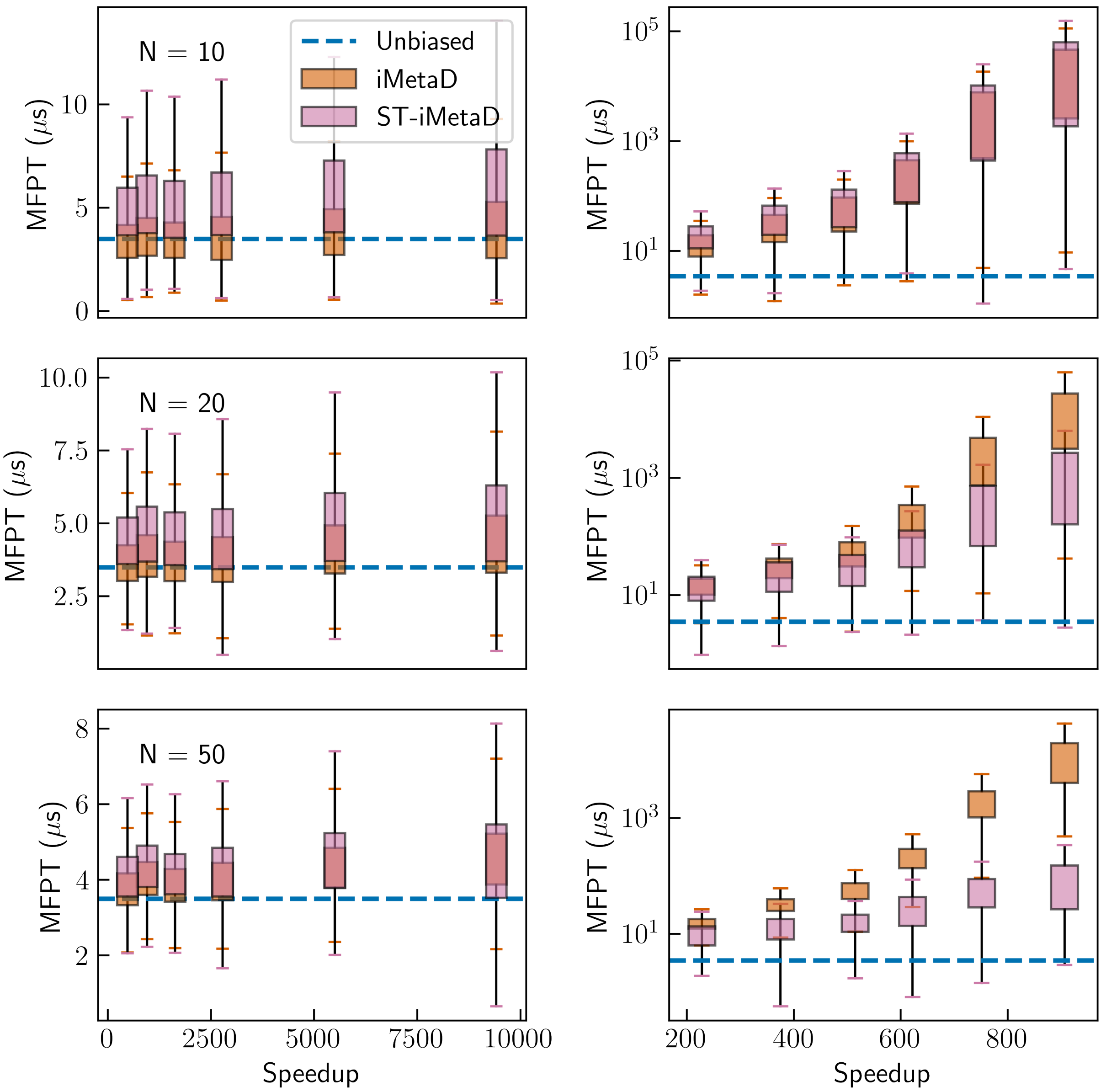

Figure S4: Results for alanine dipeptide with sets of 10 (upper row), 20 (middle row), or 50 (bottom row) trajectories in each bootstrapping batch, using the ϕ angle (left) or ψ angle (right) as CV. The blue lines show the unbiased MFPT while the boxes give estimations through standard iMetaD (orange) or ST-iMetaD (pink). The boxes show the range between the first and third quartiles and the whiskers show extreme values within 1.5 IQR below and above these quartiles.

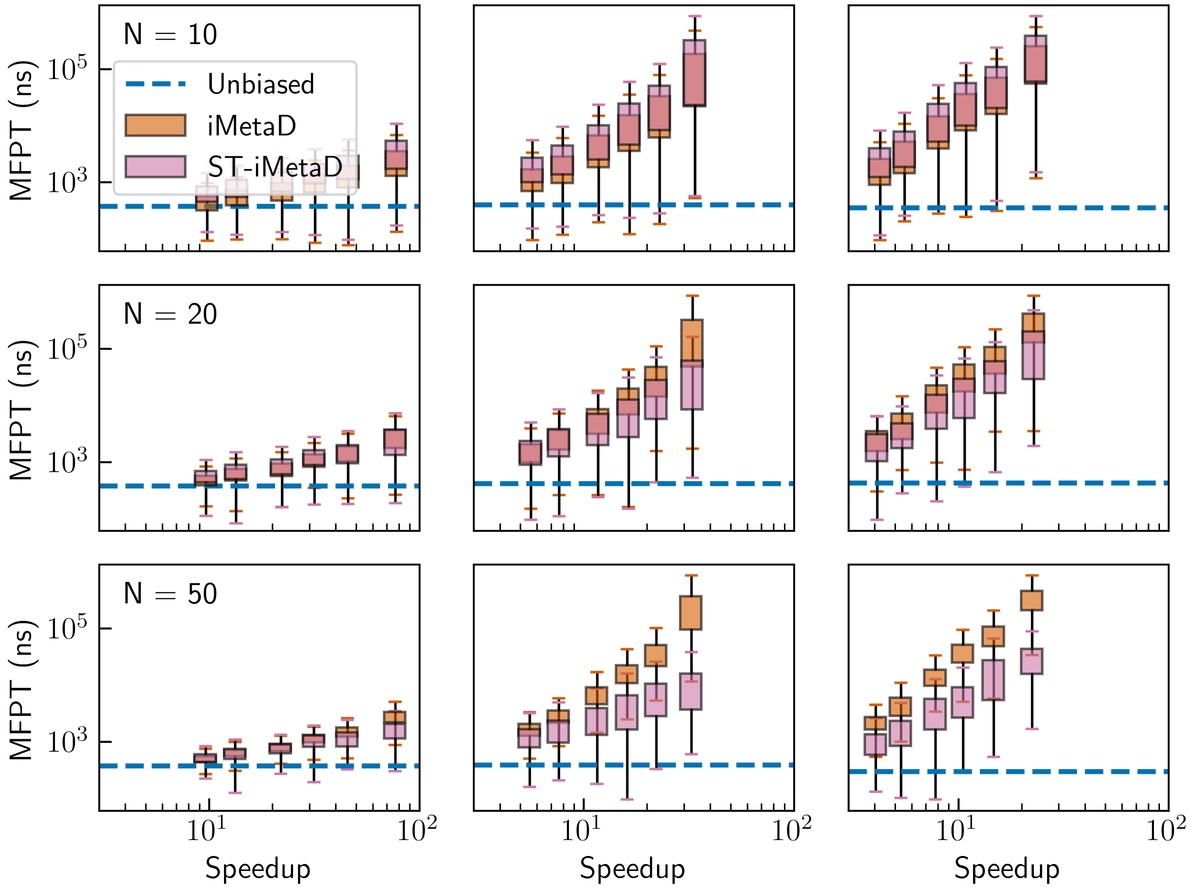

Figure S5: Results for chignolin unfolding with sets of 10 (upper row), 20 (middle row), or 50 (bottom row) trajectories in each bootstrapping batch, using CVs based on either HLDA (left column), Rg (center column), or RMSD (right column). The blue lines show the unbiased MFPT and the boxes give estimations through standard iMetaD (orange) or ST-iMetaD (pink). The boxes show the range between the first and third quartiles and the whiskers show extreme values within 1.5 IQR below and above these quartiles.

Comparison with Kramers Time-Dependent Rate

Here, we compare between ST-iMetaD and Kramers Time-Dependent Rate (KTR) for the three systems presented in the manuscript.

KTR is a recently developed kinetics inference scheme from biased simulations.[S2] Relying on Kramers theory,[S3,S4] it assumes that the kinetic rate of the biased process at time t is given by $k(t) = k_0 e^{\beta\gamma V_{MB}(t)}$, with k_0 being the unbiased kinetic rate, β the inverse temperature,

and $V_{MB}(t)$ the average maximum height of the biasing potential. k_0 and $\gamma \in [0, 1]$ are obtained from a maximum likelihood fitting to the empirical survival function.

We reproduce Figure 2, Figure 4(a), Figure 4(b), and Figure 5(b) of the manuscript, adding the MFPT estimations through KTR in red. We used open-source code provided by the authors of the method.[S5] We find that KTR underestimates the MFPT for simulations with high bias deposition rates or suboptimal CVs, as opposed to iMetaD which overestimates it. ST-iMetaD provides better predictions than KTR for the Wolfe-Quapp potential and alanine dipeptide. For Chignolin, in most CVs and bias deposition rates, ST-iMetaD provides similar accuracy to KTR, expect for the RMSD-based CV. This case suffers the most from bias over-deposition, that overestimates the MFPT, which possibly results in a fortuitous cancellation of errors for KTR.

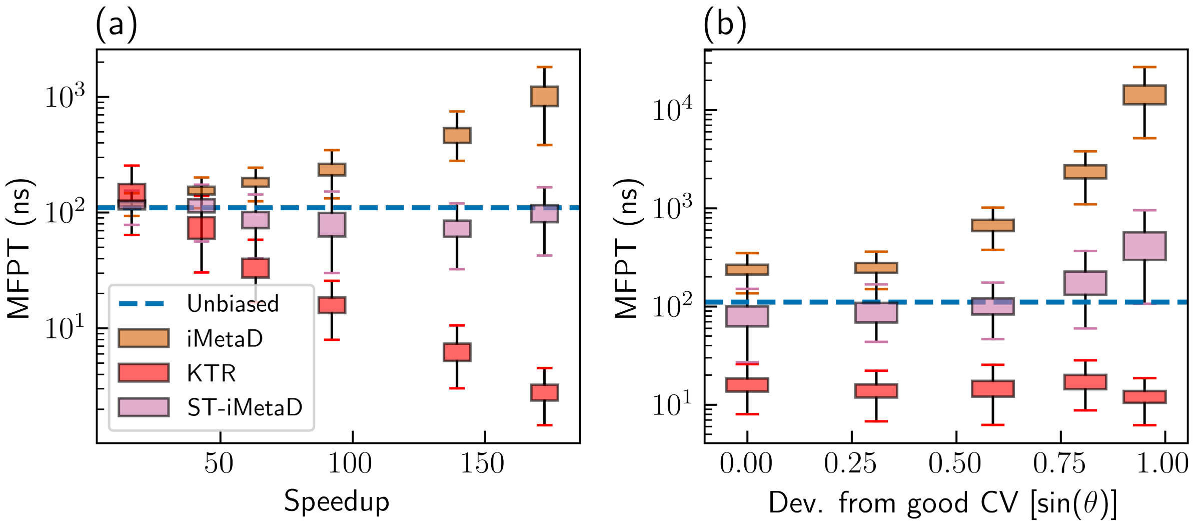

Figure S6: Results for the Wolfe-Quapp potential. (a) Estimated MFPT as a function of speedup for simulations using a good CV and different bias deposition rates from 10 to 1000 ns^{-1}. (b) Estimated MFPTs for a bias deposition rate of 200 ns^{-1}, and different choices of CV. We used either standard iMetaD (orange), KTR (red), or ST-iMetaD (pink). The boxes show the range between the first and third quartiles and the whiskers show extreme values within 1.5 IQR below and above these quartiles. The blue lines show the unbiased MFPT.

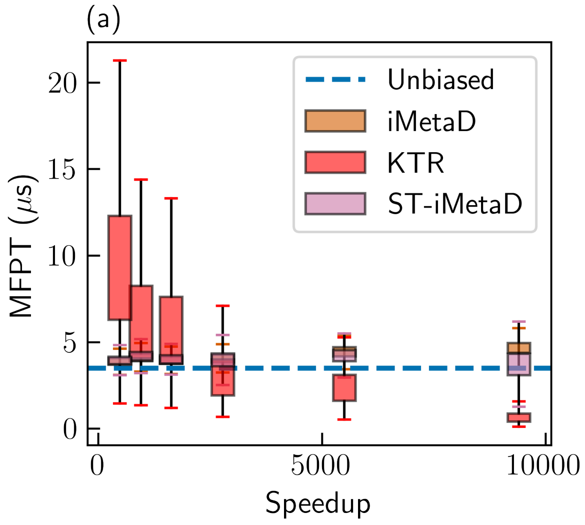

Figure S7: Results for alanine dipeptide using (a) the ϕ angle or (b) the ψ angle as CV. We used either standard iMetaD (orange), KTR (red), or ST-iMetaD (pink). The boxes show the range between the first and third quartiles and the whiskers show extreme values within 1.5 IQR below and above these quartiles. The blue lines show the unbiased MFPT.

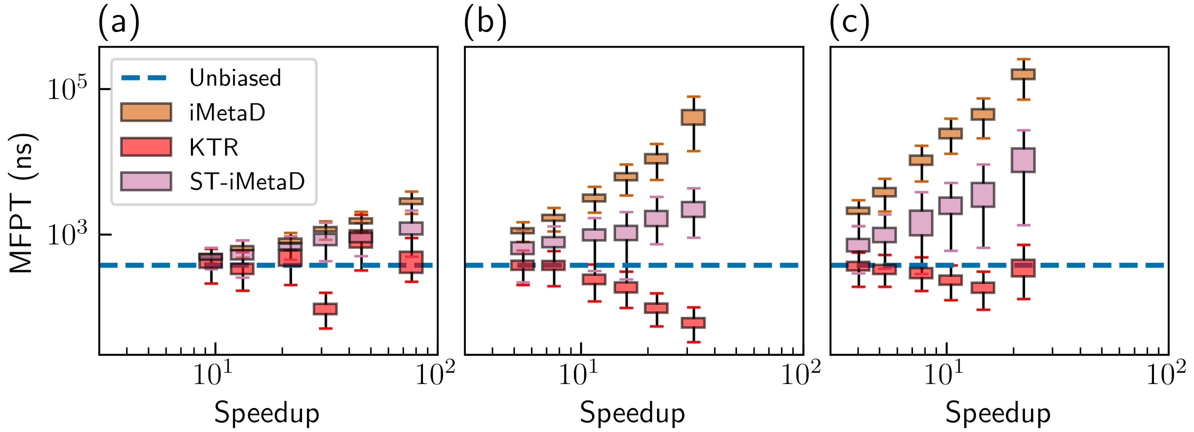

Figure S8: Results for chignolin using (a) the HLDA, (b) the Rg, or (c) the RMSD-based CVs. We used either standard iMetaD (orange), KTR (red), or ST-iMetaD (pink). The boxes show the range between the first and third quartiles and the whiskers show extreme values within 1.5 IQR below and above these quartiles. The blue lines show the unbiased MFPT.

References

(S1) Peters, B. In *Reaction Rate Theory and Rare Events Simulations*; Peters, B., Ed.; Elsevier: Amsterdam, 2017; pp 539–571.

(S2) Palacio-Rodriguez, K.; Vroylandt, H.; Stelzl, L. S.; Pietrucci, F.; Hummer, G.; Cossio, P. Transition Rates and Efficiency of Collective Variables from Time-Dependent Biased Simulations. *J. Phys. Chem. Lett.* **2022**, *13*, 7490–7496.

(S3) Kramers, H. Brownian Motion in a Field of Force and the Diffusion Model of Chemical Reactions. *Physica* **1940**, *7*, 284–304.

(S4) Peters, B. In *Reaction Rate Theory and Rare Events Simulations*; Peters, B., Ed.; Elsevier: Amsterdam, 2017; pp 435–450.

(S5) Palacio-Rodriguez, K.; Cossio, P.; Silva-Sánchez, D.; Vroylandt, H. Transition Rates, Survival Probabilities, and Quality of Bias from Time-Dependent Biased Simulations. `https://github.com/kpalaciorodr/KTR`, Accessed: March 21, 2024.
